# Supplementary figures and images for: Lawsonia intracellularis regulates nuclear factor-κB signalling pathway during infection
Source: PLoS One. 2024 Sep 26;19(9):e0310804. doi: 10.1371/journal.pone.0310804 (PMC11426430; doi:10.1371/journal.pone.0310804)

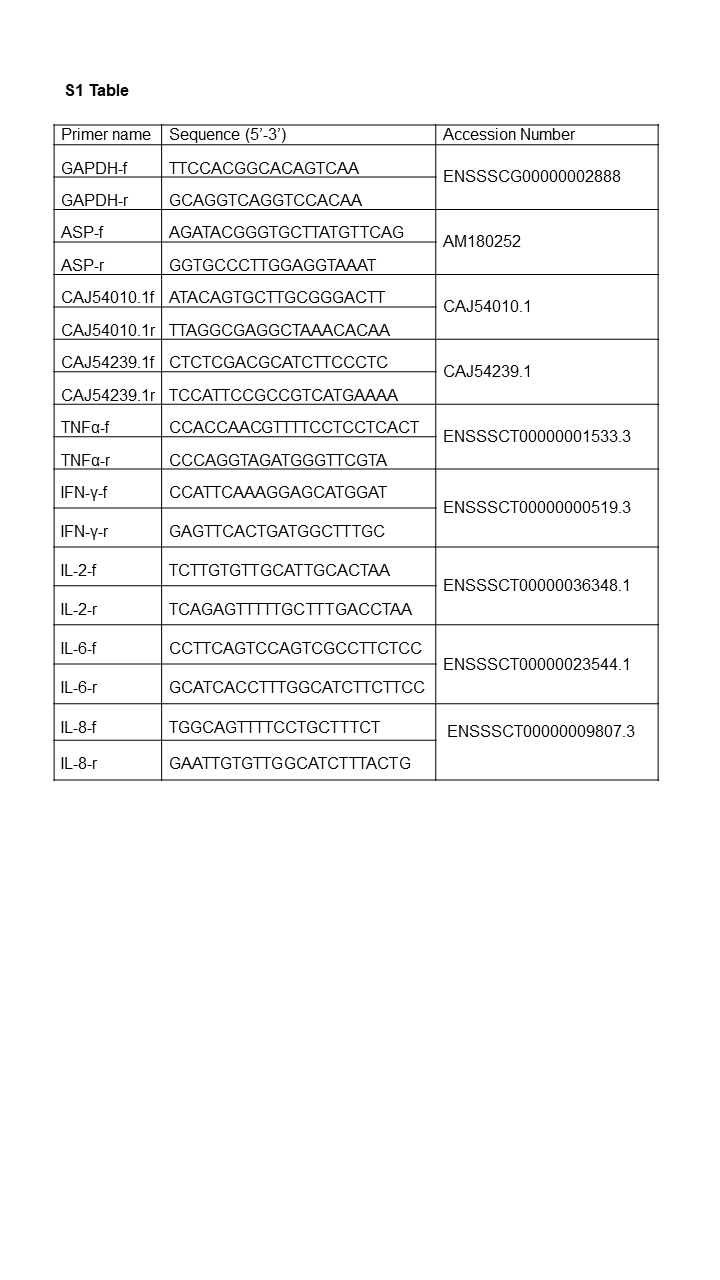

Supplement: S1 Table — (TIF) [file pone.0310804.s001.TIF]

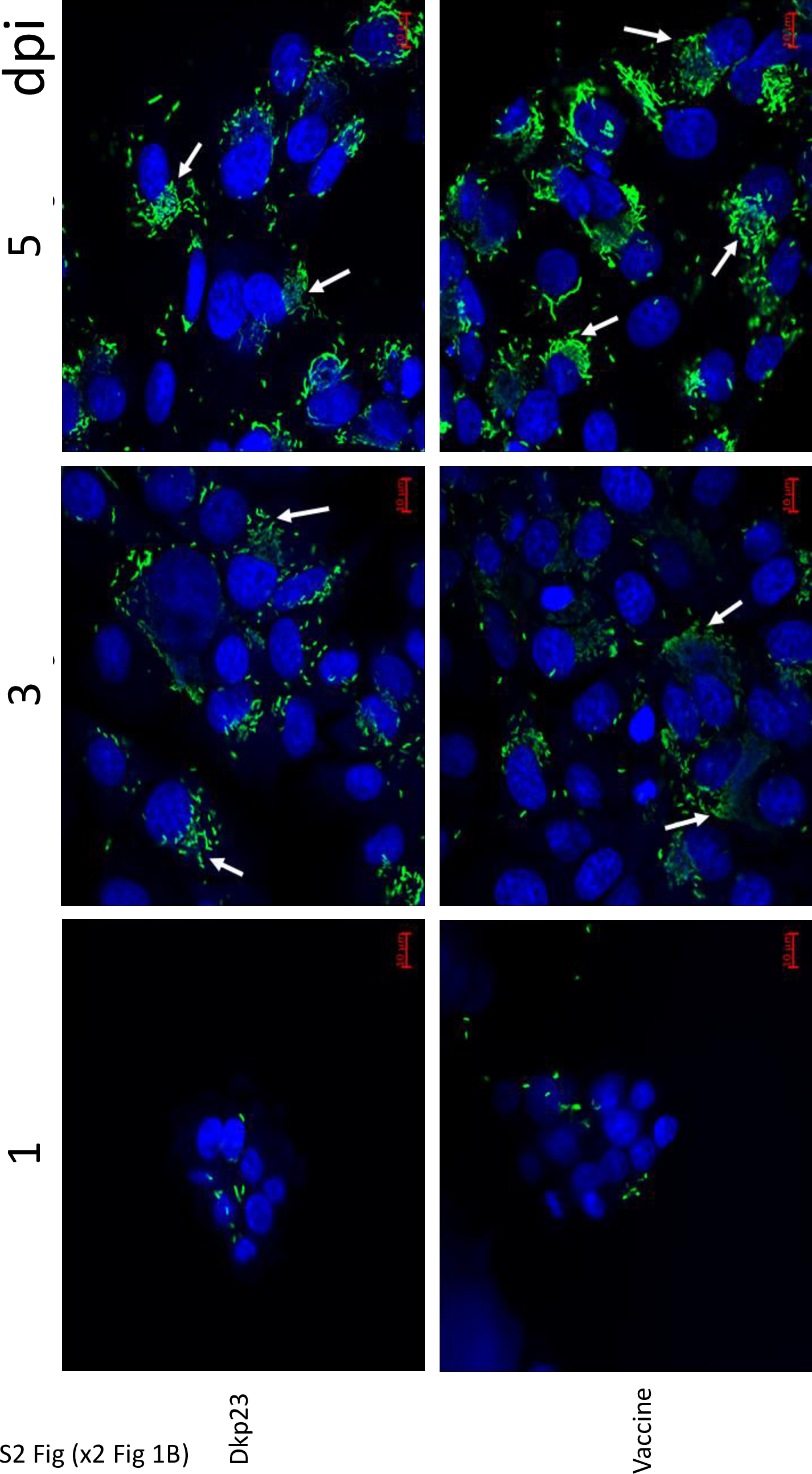

Supplement: S1 Fig — (TIF) [file pone.0310804.s002.tif]

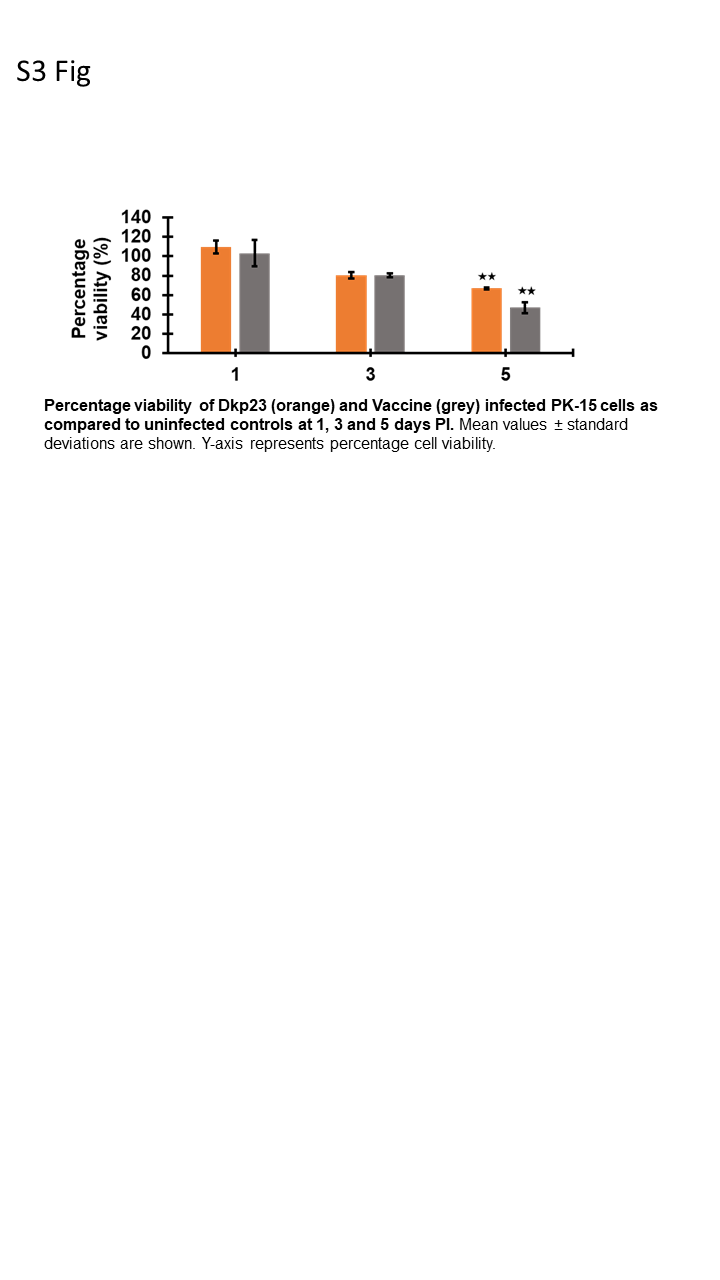

Supplement: S2 Fig — Percentage viability of Dkp23 (orange) and Vaccine (grey) infected PK-15 cells as compared to uninfected controls at 1, 3 and 5 dpi. Mean values ± standard deviations are shown. Y-axis represents percentage cell viability. (TIF) [file pone.0310804.s003.TIF]

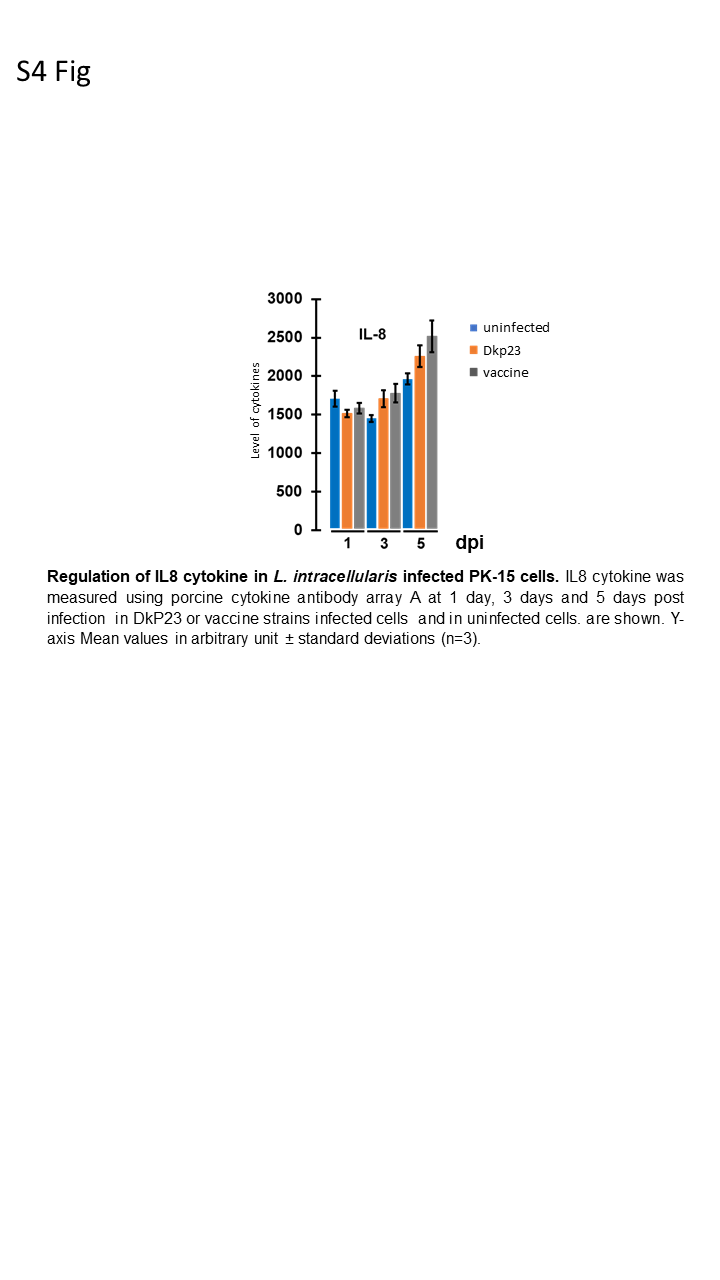

Supplement: S3 Fig — IL8 cytokine was measured using porcine cytokine antibody array A at 1, 3 and 5 dpi in Dkp23 or vaccine strains infected cells and in uninfected cells are shown. Y-axi s mean values in arbitrary unit ± standard deviations (n = 3). (TIF) [file pone.0310804.s004.TIF]

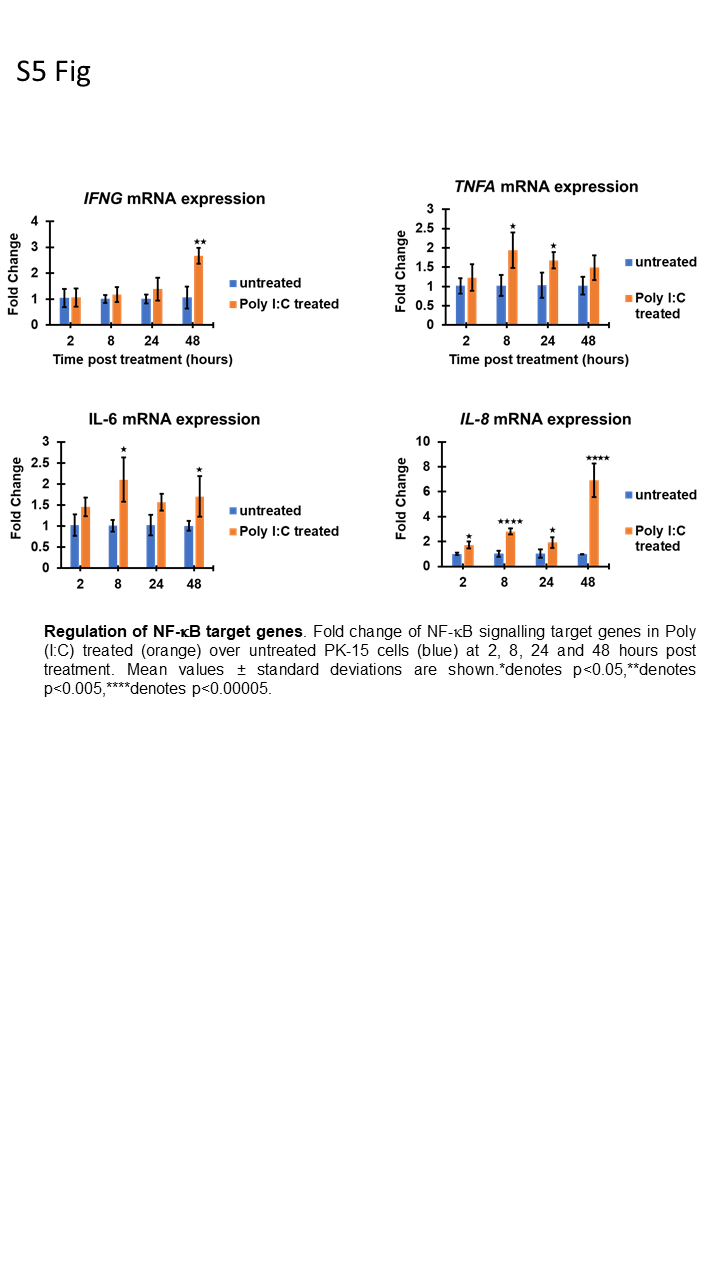

Supplement: S4 Fig — Fold change of NF-κB signalling target genes in Poly (I:C) treated (orange) over untreated PK-15 cells (blue) at 2, 8, 24 and 48 hpt. Mean values ± standard deviations are shown.*denotes p<0.05,**denotes p<0.005,****denotes p<0.00005. (TIF) [file pone.0310804.s005.TIF]

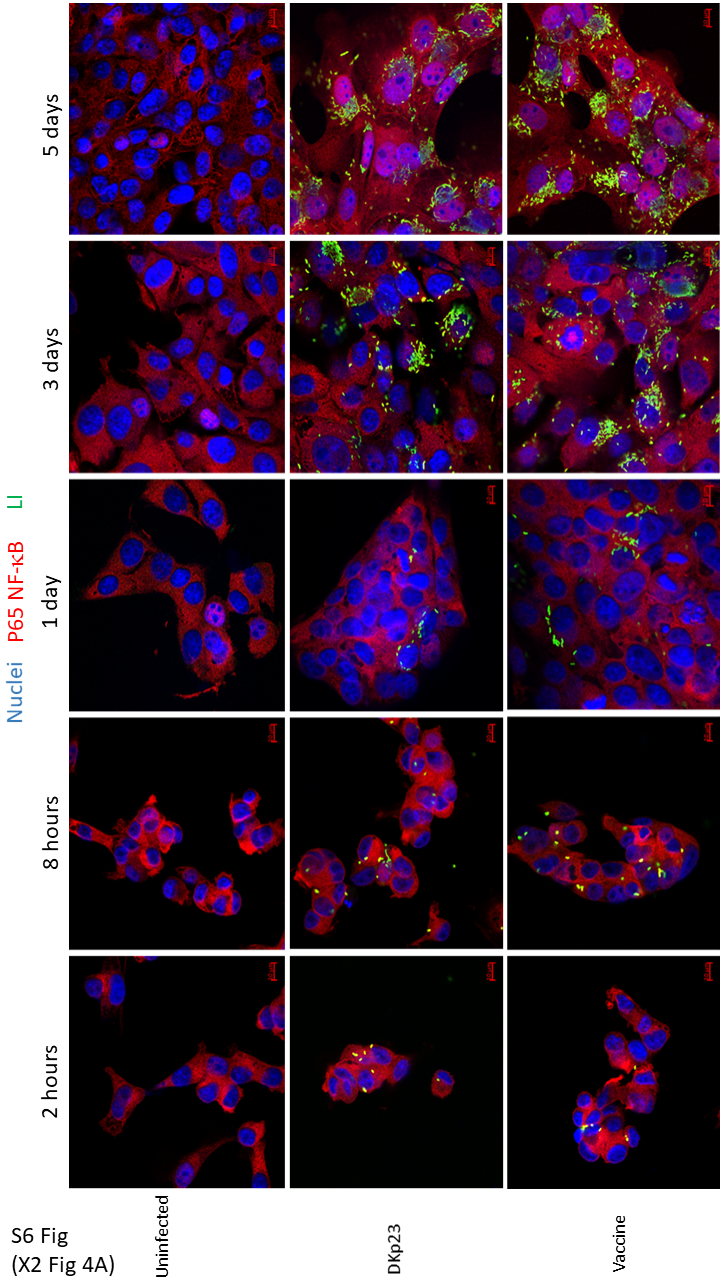

Supplement: S5 Fig — (TIF) [file pone.0310804.s006.TIF]

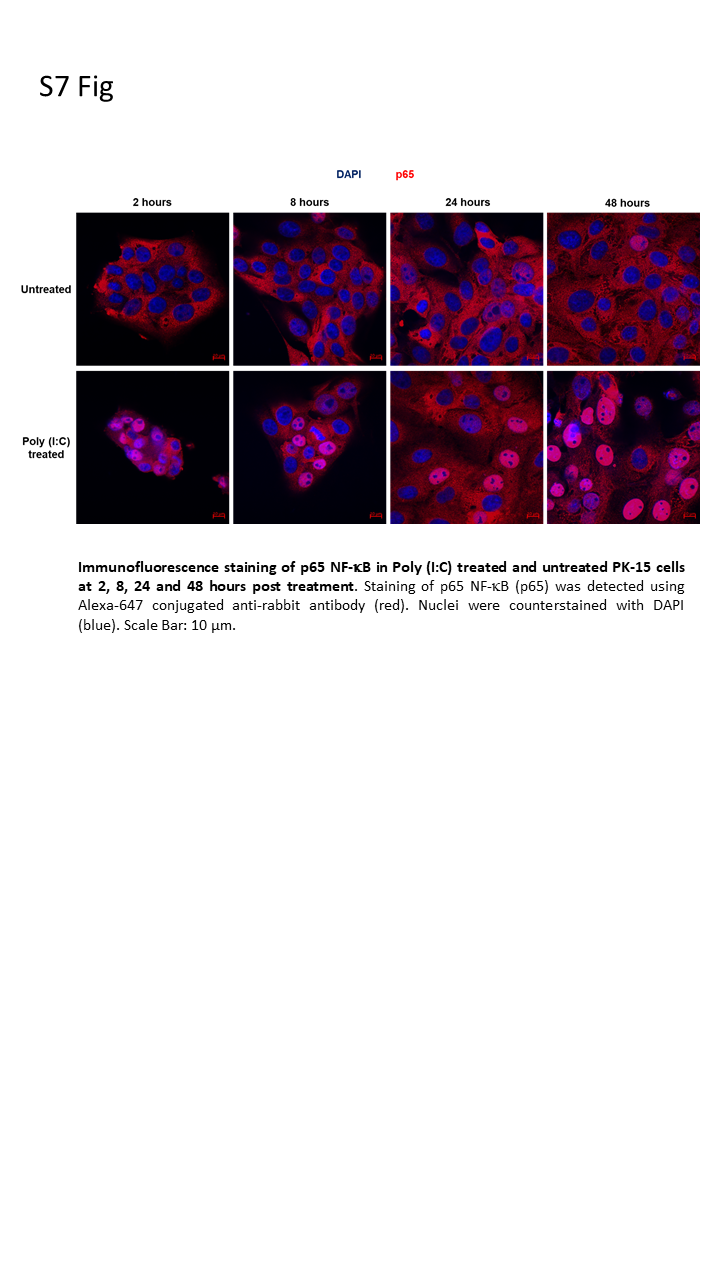

Supplement: S6 Fig — Staining of p65 NF-κB (p65) was detected using Alexa-647 conjugated anti-rabbit antibody (red). Nuclei were counterstained with DAPI (blue). Scale Bar: 10 μm. (TIF) [file pone.0310804.s007.TIF]

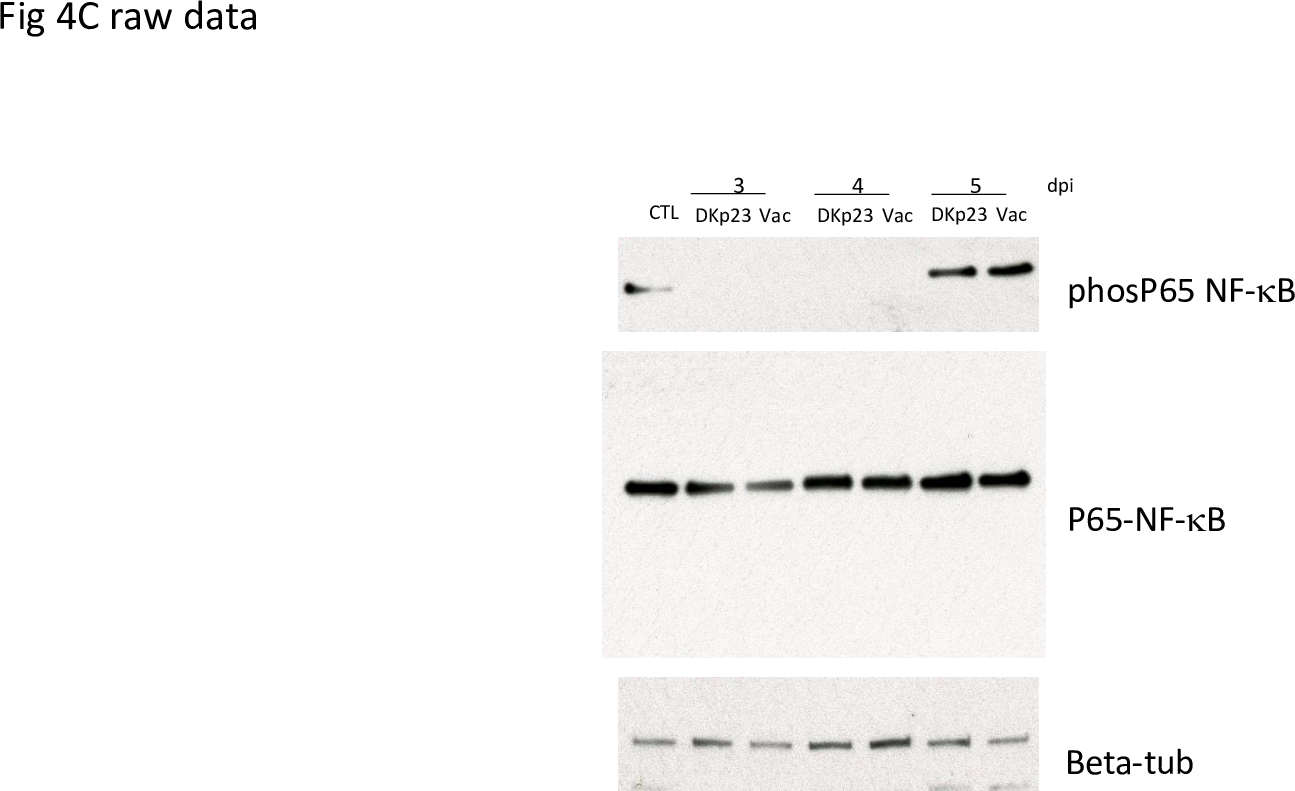

Supplement: S1 Raw images — (TIF) [file pone.0310804.s008.tif]
