# Supplementary material for: Lawsonia intracellularis regulates nuclear factor-κB signalling pathway during infection
Source: PLoS One. 2024 Sep 26;19(9):e0310804. doi: 10.1371/journal.pone.0310804 (PMC11426430; doi:10.1371/journal.pone.0310804)
Supplement: Attachment — Submitted filename: 2024-reviewer reports.docx [file pone.0310804.s009.docx]

The authors investigated the nuclear factor-κB (NF-κB)- regulated immune response against infection of a clinical strain Dkp23 and a live-attenuated Enterisol vaccine strain in PK-15 cells. They found that expression of NF-κB target genes TNF-α, IFN-γ, IL-6 and IL-8 were modulated during the course of infection. They also found that there was a significant increase in p65 NF-κB activation, including protein nuclear translocation and phosphorylation in *L. intracellularis* infected cells at 5 dpi. The methods were described clearly. However, I still have some comments.

Major comments:

1. In Figure 4C, authors detected the expression of phos-p65, p65 and β-tubulin in untreated and *L. intracellularis* infection cells. The basal expression of p-p65 was detected in the untreated cells, but completely undetectable in the *L. intracellularis* infected cells, at 3 and 4 dpi. Why the expression levels of p-p65 were not detected at 2, 8 hpi, and 1 dpi, as in the previous experiments? Is there a possibility that NF-κB signaling pathway was inhibited to facilitate the bacterium replication in the cytoplasm during the early phase of *L. intracellularis* infection, while NF-κB signaling pathway was activated to induce an inflammatory response, which aiding the bacterium escape the cell during the late phase infection. Additionally, besides detecting p-p65, can the levels of p65 in the cytoplasm and nucleus be simultaneously detected to demonstrate NF-κB activation?

2. In Figure 5B, authors shows that the *L. intracellularis* bacterial load was significantly reduced upon TNF-α treatment when PK-15 cells were challenged with either Dkp23 or the vaccine strain. TNF-α was added 1 d after *L. intracellularis* infection and the bacterial colonization was detected after 4 d of infection. During early infection, *L. intracellularis* likely needs to inhibit the host's inflammatory response to aid its proliferation in host cells. Therefore, the addition of TNF-α, activating the NF-κB signaling pathway, inhibits its colonization in the cells. This is consistent with the results in Figure 4C and can be further discussed.

Minor comments:

1. It is recommended to reduce the number of keywords.

2. Line 22, I believe 'PI' should be written as 'dpi'. Please, check this.

3. In some places throughout the text, a space is missing between the end of the subordinate clause and the principal clause. Please, check this. (incl. lines 23, 78, 213, 288).

4. Throughout the text, some time units have full names, and some don't. Please, make sure this is consistent throughout.

5. Line 25, I believe "PK-15" should be " PK-15 cells". Please check it.

6. Line 104-105: I believe "100 units/100 μg per mL " should be "100 units ml^−1^". Please check it.

7. There are no spaces between numbers and units in the text. Please, check this. (incl. lines 120, 126, 127, 130, 139, 150, 159, 168, 194, 196, 200, 253, 255, 301, 312)

8. Please list the reference (incl. Line 128, 131)

9. I believe "days PI" should be "dpi". Please check it. (incl. line 151, 179, 188, 204, 254, 299, 303, 417,)

10. Line 269, I believe " DPI " should be "dpi". Please check it.

11. I believe " P65 " should be "p65". Please check it. (incl. line 297, 302, 306)

12. Line 314, I believe "P cell " should be "PK-15 cell". Please check it.

13. Line 322: I believe "qRT-PCR " should be "qPCR". Please check it.

14. I believe " DKp23 " should be "Dkp23". Please check it. (incl. line 307, 315)

15. I believe " TNFα " should be "TNF-α". Please check it. (incl. line 16, 259, 349)

16. Line 272: I believe "PK15 " should be "PK-15". Please check it.

17. I believe " IFN-*ϒ*" should be "IFN-γ". Please check it. (incl. line 262, 264)

18. Line 423, I believe " NF-KB " should be "NF-κB". Please check it.

19. Line 428, I believe " NF-kB " should be "NF-κB". Please check it.
